# Oqtans: the RNA-seq workbench in the cloud for complete and reproducible quantitative transcriptome analysis

**DOI:** 10.1093/bioinformatics/btt731

**Published:** 2014-01-11

**Authors:** Vipin T. Sreedharan, Sebastian J. Schultheiss, Géraldine Jean, André Kahles, Regina Bohnert, Philipp Drewe, Pramod Mudrakarta, Nico Görnitz, Georg Zeller, Gunnar Rätsch

**Affiliations:** ^1^Computational Biology Center, Memorial Sloan-Kettering Cancer Center, New York, NY, USA, ^2^Machine Learning in Biology Group, Friedrich Miescher Laboratory, Tübingen, Germany, ^3^LINA, Combinatorics and Bioinformatics Group, University of Nantes, Nantes, France, ^4^Machine Learning/Intelligent Data Analysis Group, Technical University, Berlin, Germany and ^5^Structural and Computational Biology Unit, European Molecular Biology Laboratory, Heidelberg, Germany

## Abstract

We present *Oqtans*, an open-source workbench for quantitative transcriptome analysis, that is integrated in *Galaxy*. Its distinguishing features include customizable computational workflows and a modular pipeline architecture that facilitates comparative assessment of tool and data quality. *Oqtans* integrates an assortment of machine learning-powered tools into *Galaxy*, which show superior or equal performance to state-of-the-art tools. Implemented tools comprise a complete transcriptome analysis workflow: short-read alignment, transcript identification/quantification and differential expression analysis. *Oqtans* and *Galaxy* facilitate persistent storage, data exchange and documentation of intermediate results and analysis workflows. We illustrate how *Oqtans* aids the interpretation of data from different experiments in easy to understand use cases. Users can easily create their own workflows and extend *Oqtans* by integrating specific tools. *Oqtans* is available as (i) a cloud machine image with a demo instance at cloud.oqtans.org, (ii) a public *Galaxy* instance at galaxy.cbio.mskcc.org, (iii) a *git* repository containing all installed software (oqtans.org/git); most of which is also available from (iv) the *Galaxy Toolshed* and (v) a *share string* to use along with *Galaxy CloudMan*.

**Contact:** vipin@cbio.mskcc.org, ratschg@mskcc.org

**Supplementary information:** Supplementary data are available at *Bioinformatics* online.

## 1 INTRODUCTION

Technological advance in large-scale sequencing has revolutionized molecular biology. Its application to profiling the transcriptome, the total complement of cellular RNA, called RNA-seq, provides an unmatched dynamic range for expression quantification and single base pair resolution for the discovery of new transcripts (Mortazavi *et al.*, 2008). However, analyzing these complex data to their full potential requires computational frameworks.

Here, we present Oqtans, the online platform for quantitative RNA-seq data analysis (online since 2010). Its integration into the Galaxy framework ensures transparent and reproducible computational analyses. *Oqtans* provides a *Galaxy* interface to many recently developed RNA-seq analysis tools, and this way considerably extends the standard repertoire of the *Galaxy* toolbox (usegalaxy.org). To reach non-expert users and experienced developers, we provide the *Oqtans* tool suite in five incarnations: (i) as a cloud machine image (see cloud.oqtans.org for a demo), (ii) as a public *Galaxy* instance at galaxy.cbio.mskcc.org, (iii) as a *git* repository (oqtans.org/git); most of these tools are moreover available from (iv) the *Galaxy Toolshed* and (v) a preconfigured *share string* to launch Galaxy CloudMan using sharing instance functionality.

## 2 RESULTS

*Oqtans* provides a versatile analysis workbench for RNA-seq data comprising tools suitable for basic and advanced analysis tasks (see Supplementary Table S1 for a current list of *Oqtans* tools and Supplementary Table S2 for supported file formats). Their modular organization within the *Galaxy* framework allows advanced users to easily customize and extend analysis workflows.

We showcase *Oqtans* capabilities in use cases for which all data, parameters, intermediate output and final results are made public on a *Page* in our *Galaxy* cloud instance (see oqtans.org/usecases).

**Fig. 1.**
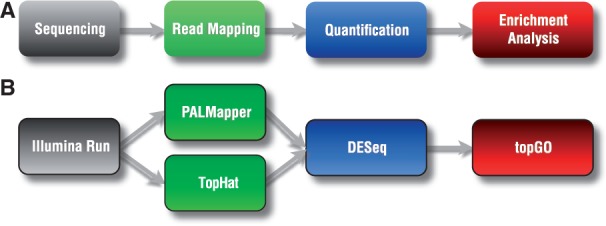
Schematic workflows of the *Oqtans* use cases. (**A**) The general steps needed to perform the analysis. (**B**) Tools included in *Oqtans* used for differential expression and GO term enrichment analysis (use case 1). The same workflow in the *Galaxy* instance is shown in Supplementary Figure S1

As a first use case, we wanted to identify annotated genes that were differentially expressed between male and female *Drosophila melanogaster* fruit flies [using data from (Daines *et al.*, 2011)]. This analysis requires three major steps: read alignment, quantification and enrichment analysis (Fig. 1A and B). The chosen *Oqtans* tools were combined in a workflow (Supplementary Fig. S1).

After starting an *Oqtans* cloud instance in Amazon Web Service EC2 (machine image ami-65376a0c) and importing the RNA-seq read data from the NCBI short read archive, we aligned these to the reference genome. *Oqtans* currently offers three tools for spliced alignments of short reads, *Tophat* (Trapnell *et al.*, 2009), *STAR* (Dobin *et al.,* 2013) and *PALMapper* (Jean *et al.*, 2010). Subsequently, we determined genes that were differentially expressed in males and females using *DESeq*, which tests read counts for statistically significant differences (Anders and Huber, 2010).

To determine enriched Gene Ontology (GO) terms among differentially expressed genes, we supplied the gene list to *topGO* (Alexa *et al.*, 2006), which we integrated into *Oqtans*. Its graphical output highlights expression differences in genes annotated with the functions ‘reproduction’ and ‘sex determination’, as is expected for this comparison between male and female fruit fly transcriptomes (see Supplementary Fig. S3).

The whole experiment excluding short read alignment requires ∼10 min of compute time. Duration of the alignment depends on the number and size of compute nodes that can be allocated for this task (20 min in our setup with 19 ‘4× large memory’ instances on Amazon Web Service).

**Fig. 2.**
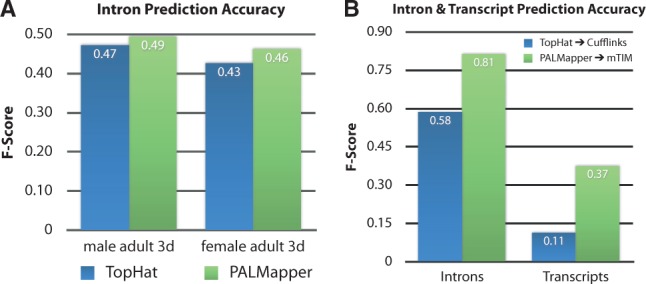
(**A**) Performance comparison of two alignment programs integrated in *Oqtans*, evaluated on the data from the use case in terms of intron accuracy (see Supplementary Fig. S3 for details). Such comparative evaluations are made easy, since the replicability assertion of the *Galaxy Oqtans* setup ensures otherwise identical comparisons. (**B**) Performance comparison from Görnitz et al. (2011), where PALMapper and TopHat alignments are processed with the *de novo* transcript inference tools *mTIM* and *Cufflinks*, again demonstrating the value of *Oqtans* for comparisons of analysis tool

Uniquely within *Oqtans* and through the benefits of the *Galaxy* framework, we can directly compare integrated software tools on the same input data. This is of great value for a researchers who are looking for the most appropriate and accurate algorithm to analyze their newly generated data. For instance, for *de novo* transcript prediction, the accuracy of the alignments is particularly important. We demonstrate this in a comparison of the accuracy of introns predicted from spliced alignments against the genome annotation generated by *TopHat* and *PALMapper* (Fig. 2A and see Supplementary Section S3 for details). Although alignment accuracy may have a negligible effect on the detection of differentially expressed annotated genes, it becomes crucial for *de novo* inference of transcripts (isoforms). Owing to the high resolution provided by RNA-seq, the discovery of novel transcript isoforms from these data has been a prime analysis goal. In Görnitz *et al.* (2011), the authors compared the accuracy of transcript inference by combining different read alignment programs (PALMapper, TopHat) with different transcript predictors (margin-based Transcript Identification Method, Cufflinks). All tools used in this example are integrated into *Oqtans* and can be easily combined in workflows to reproduce this and similar comparisons (Fig. 2B) (see Section S3 at Supplementary Material for more details).

## 3 DISCUSSION

As high-throughput genome and transcriptome sequencing becomes routine in many laboratories around the world, there is an increasing demand for standardized data analysis. Directly associated with this need are accessibility, transparency and persistency of analysis pipelines. As a *Galaxy* web server, *Oqtans* brings us closer to these goals (Schultheiss, 2011) for the important task of RNA-seq data analysis by providing easy access to state-of-the-art analysis tools to a wide audience. Importantly, while profiting from many free software development efforts, its user friendly interface abstracts from programming languages and operating systems, and thus enables even inexperienced users to rapidly analyze their RNA-seq data.

## Supplementary Material

Supplementary Data
