# Supplementary material for: Oqtans: the RNA-seq workbench in the cloud for complete and reproducible quantitative transcriptome analysis
Source: Bioinformatics. 2014 Jan 11;30(9):1300–1. doi: 10.1093/bioinformatics/btt731 (PMC3998122; doi:10.1093/bioinformatics/btt731)
Supplement: Supplementary Data [file supp_30_9_1300__index.html]

Oqtans: The RNA-seq Workbench in the Cloud for Complete and Reproducible Quantitative Transcriptome Analysis — Oqtans: the RNA-seq workbench in the cloud for complete and reproducible quantitative transcriptome analysis — Oqtans: the RNA-seq workbench in the cloud for complete and reproducible quantitative transcriptome analysis — Supplementary Data 

# Oqtans: the RNA-seq workbench in the cloud for complete and reproducible quantitative transcriptome analysis

## Supplementary Data

files

**Files in this Data Supplement:**

- Supplementary Data - pdf file
