# Supplementary material for: Oqtans: the RNA-seq workbench in the cloud for complete and reproducible quantitative transcriptome analysis
Source: Bioinformatics. 2014 Jan 11;30(9):1300–1. doi: 10.1093/bioinformatics/btt731 (PMC3998122; doi:10.1093/bioinformatics/btt731)
Supplement: Supplementary Data [file supp_btt731_supplemental_material.pdf]

Supplementary Material:
Oqtans: The RNA-seq Workbench in the Cloud for Complete and Reproducible Quantitative Transcriptome Analysis

Vipin T. Sreedharan[1,2], Sebastian J. Schultheiss[2], Géraldine Jean[2,3], André Kahles[1,2], Regina Bohnert[2], Philipp Drewe[1,2], Pramod Mudrakarta[2], Nico Görnitz[4], Georg Zeller[5,2], and Gunnar Rätsch[1,2]

[1]Computational Biology Center, Memorial Sloan-Kettering Cancer Center, New York, USA
[2]Machine Learning in Biology Group, Friedrich Miescher Laboratory, Tübingen, Germany
[3]LINA, Combinatorics and Bioinformatics Group, University of Nantes, Nantes, France
[4]Machine Learning and Intelligent Data Analysis Group, Technical University Berlin, Berlin, Germany
[5]Structural and Computational Biology Unit, European Molecular Biology Laboratory, Heidelberg, Germany

Abstract

We present *Oqtans*, an open-source workbench for quantitative transcriptome analysis, that is integrated in the *Galaxy* framework. Its distinguishing features include customizable computational workflows and a modular pipeline architecture that facilitates comparative assessment of tool and data quality.

Oqtans integrates, for the first time, an assortment of sophisticated machine learning-powered tools into *Galaxy*, that show superior or equal performance to state-of-the-art tools. Implemented tools comprise of a complete transcriptome analysis workflow: short-read alignment, transcript identification/quantification, and differential expression analysis. Moreover, *Oqtans* is scalable in the cloud in terms of data storage and computing time needs. Finally, *Oqtans* and *Galaxy* facilitate persistent storage, data exchange, and documentation of intermediate results and analysis workflows. We illustrate how *Oqtans* aids the interpretation of data from different experiments in easy to understand *use cases*. Users can easily create their own workflows and extend *Oqtans* by integrating specific tools. *Oqtans* is available as

- **(a)** a cloud machine image with a demo instance available at `cloud.oqtans.org`,
- **(b)** a public *Galaxy* instance at `galaxy.cbio.mskcc.org` and
- **(c)** a *git* repository containing all installed software at `oqtans.org/git` most of which is also avaliable from
- **(d)** the *Galaxy Toolshed* (`bioweb.me/gxtoolshed`) and
- **(e)** a *share string* cm-ba5c56b95144e564f70e5762dc5fa177/shared/2013-11-07–22-16/ to ditribute cluster-sharing functionality directly from *Galaxy CloudMan* launch.

1 Introduction

The majority of RNA-seq analyses require four essential steps: sequencing, read mapping, transcript prediction, and quantification. The sheer number of different software programs available for the same task can be overwhelming. For instance, today, roughly a dozen tools have been published that specifically align RNA-seq reads to a reference genome and take into account or detect novel splicing events (PALMapper (Jean *et al.*, 2010; De Bona *et al.*, 2008), TopHat (Trapnell *et al.*, 2009), MapSplice (Wang *et al.*, 2010), SpliceMap (Au *et al.*, 2010), etc.), and there are likely many more tools for this purpose. It is difficult for researchers to determine which ones are best suited for their experimental setup. The difficulty is to first find the most accurate or appropriate program for each task and second to combine several programs effortlessly to obtain a complete pipeline.

2 Availability

2.1 Availability of the *Oqtans*-enabled images

We have extended a virtual machine image that can be used with the tools we have created. These tools are released under an open-source license (GPL). The machine image we used is available publicly (as "`ami-65376a0c`") from Amazon Web Services (AWS) and can be launched directly in an EC2 environment. The following basic steps are required to create an new *Oqtans* instance in the Amazon EC2 cloud: (a) create an account with AWS (e.g., a free tier account) and obtain security credentials, (b) use the "Request Instances Wizard" and create an instance based on an *Oqtans* image (i.e., `ami-65376a0c` & instance type `m1.large`), (c) enter security credentials as "User Data" for the new instance, (d) define access rules and allow http access, and finally, (e) launch the instance. The instance will shortly be available with a ready-to-use *Galaxy* server. Then you can (f) execute the *Oqtans* setup script. Detailed instructions are available at `oqtans.org/instantiate`.

Cloud service providers provide persistent storage of results, a service invaluable for science: once an analysis for a publication is complete, the entire machine image and all dependent data files can be archived, ready to be run again with all original data and parameter settings in place. We found it easiest to create a fresh instance with each project, that can be independently archived, removed, or distributed. Persistence, in this case, is limited by the contract of the cloud provider to the one paying for it. To ensure scientific reproducibility, it should be broadly explored, how data from publicly-funded research is best made publicly accessible in a sustainable manner.

2.2 Installation of *Oqtans* tools and images

The current version of *Oqtans* can be downloaded from our public git repository `git@github.com:ratschlab/oqtans.git` into an existing *Galaxy* cloud instance or a local *Galaxy* installation. To enable the tools, users have to include the tool description into the `tool_conf.xml` file of the running instances (for detailed instructions, see `oqtans.org/install`).

The *Oqtans* tools are also available individually via the *Galaxy* toolshed `toolshed.g2.bx.psu.edu`. Upcoming versions of *Galaxy* and the toolshed will allow fully automatized installation and integration of new tools (personal communication, *Galaxy* Developer Team). Once the *Galaxy* toolshed is fully operational, we will provide versions of the tools that automatically install within a running *Galaxy* instance.

3 Evaluations in Figure 2

From the Short Read Archive (SRA) we downloaded reads with accessions SRX019652, the three days old female adults, and SRX019653, the three days old male adults, of a *D. melanogaster* wild type strain commonly used in laboratories, called Canton-Special. The data consist of two sets of around 25 millions (female) and 15 millions (male) 75 bp paired-end reads generated with the Illumina Genome Analyzer II. We used two short-read alignment programs to align the paired-end, spliced reads, namely *Tophat* (Trapnell *et al.*, 2009) version 1.1.4 and *PALMapper* version 0.4 (Jean *et al.*, 2010). We have used the flybase annotation (**?**) together with the *evaluation-tool* described in Jean *et al.* (2010) to estimate the intron prediction accuracy (sensitivity and specificity was computed and used to compute the displayed F-Score). A new version of this tool is also available on our *Galaxy* instance `galaxy.cbio.mskcc.org` (section "NGS: Evaluation", tool "Compare Spliced Alignment to Annotation").

To generate Figure 2b we used a *C. elegans* dataset and followed the same steps as in Figure 2b of Görnitz *et al.* (2011a) also comparing *Cufflinks* and *mTIM*. A major difference is that in Görnitz *et al.* (2011a) we used the same alignments for both methods, whereas here we use *Tophat* alignments for *Cufflinks* and *PALMapper* alignments for *mTIM*.

4 Supplementary Use Case: Gene family expression in *Arabidopsis thaliana*

In the second use case, we computed and visualized fractions of unexpressed, expressed, and differentially expressed gene families. Different gene families often behave differently when comparing the expression levels of two natural accessions (strains) from the same species. In this example, we examined two strains from the model plant *Arabidopsis thaliana*. This example is taken from the study of genomes and transcriptomes of multiple *Arabidopsis* strains (Gan *et al.*, 2011) that compared the reference sequence Col-0 (Columbia) to the accession known as Can-0 (Canary Islands). The latter accession comes from a population that was isolated for a long time and shows many differences to the reference sequence.

Comparing lists of differentially expressed genes among different strains of the same species leads to interesting biological insights. For example, in different *Arabidopsis* accessions, the genes encoding the plants' "immune system" (pathogen defense and production of glucosinolates to deter herbivores) are the most differentially expressed group. For accessions that are found at different latitudes around the globe as it is the case in our example, genes associated with flowering time show stark contrasts. As mentioned in Gan *et al.* (2011), we expect striking expression polymorphisms for the type II MADS box transcription factor family, which includes genes specific to flowering, whereas housekeeping genes are much more constant across different accessions.

The entire pipeline for this comparison consists of aligning short reads, quantifying them, testing for differential expression, assigning genes to their families, and visualizing the result (Figure 1). We downloaded the aligned read data from the resources website of the 19 genomes of *Arabidopsis thaliana* project by Gan *et al.* (2011) (`bioweb.me/19g`) for the accessions Col-0 and Can-0. In total, between 1,241,437 and 4,920,935 reads had been aligned with PALMapper Jean *et al.* (2010). That were use for *in silico* quantification.

With DESeq, which we integrated into in *Oqtans*, we counted the number of reads mapping within each unique exonic region of the genes in the TAIR annotation Lamesch *et al.* (2012) that mapped to the accessions' genome coordinates. We also used DESeq to test for differential

expression of all 65,238 annotated features (i.e., genes, pseudogenes, transposable elements and others) between the two accessions of interest.

Employing the conservative Bonferroni correction for multiple testing, we obtained an adjusted p-value for differential expression of each gene. From the TAIR database (Lamesch *et al.*, 2012), we downloaded information about gene names and their families. Finally, we applied GeneSetter to display the fractions of expressed, differentially expressed and non-expressed genes per family (see Supplementary Figure S2, which is very similar to Figure 4B in Gan *et al.* (2011)). With our tool GeneSetter (Supplementary Table S1), gene lists with meta information that are proper subsets, differences, and complements of one another can be plotted. The figures created are versatile visualizations of the annotation and the corresponding differences in the lists. Examples include the overrepresentation of transcription factor binding sites in regulatory regions of a gene, as they are used within KIRMES Schultheiss *et al.* (2009), or genes that have a certain GO term in common, for instance from the first use case.

5 Supplementary Figures and Tables

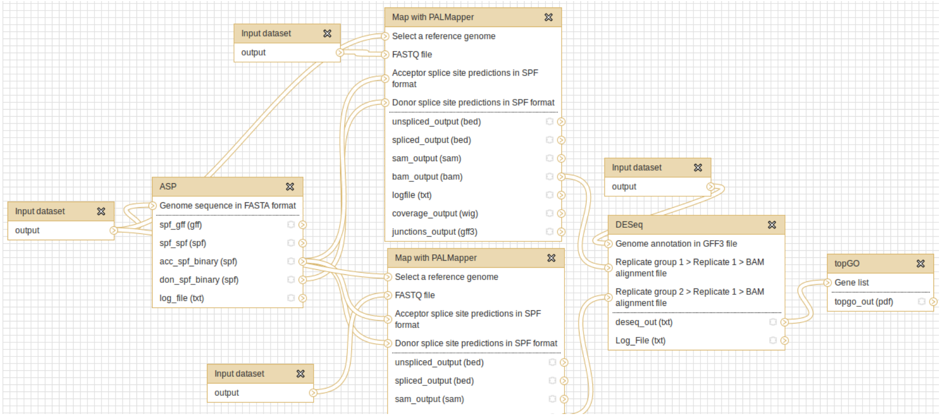

Supplementary Figure S 1: The workflow of the first use case as it is represented in the *Oqtans Galaxy* instance.

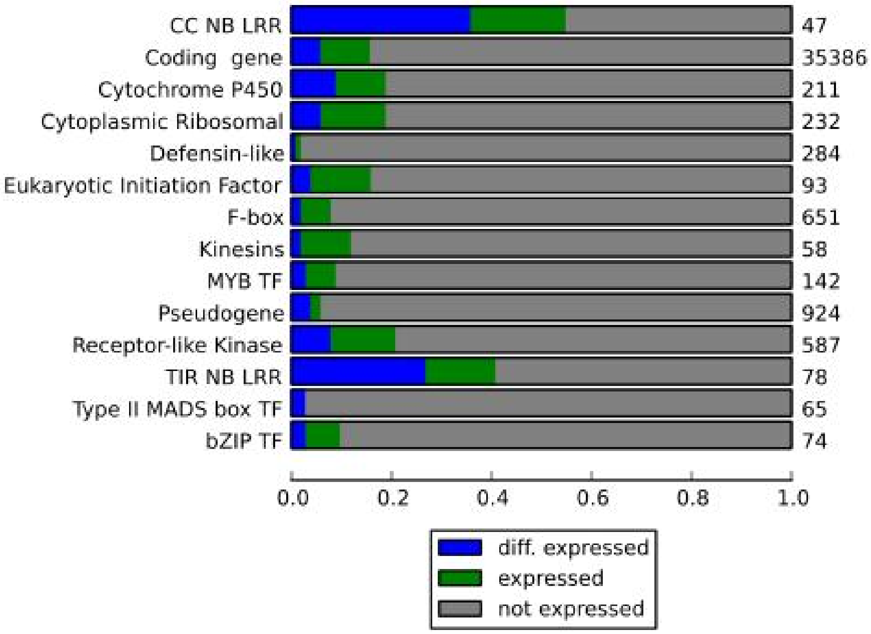

Supplementary Figure S 2: Output of GeneSetter for the second use case showing the fraction of differentially expressed genes, expressed genes, and genes that are not expressed. Gene expression varies, often strongly, by category or gene family. The numbers on the right in each row are gene counts, i.e., the size of the gene lists. The figure is generated by running the GeneSetter with the following input data: lists of expressed and differentially expressed genes (output of DESeq) as well as a table mapping genes to gene families.

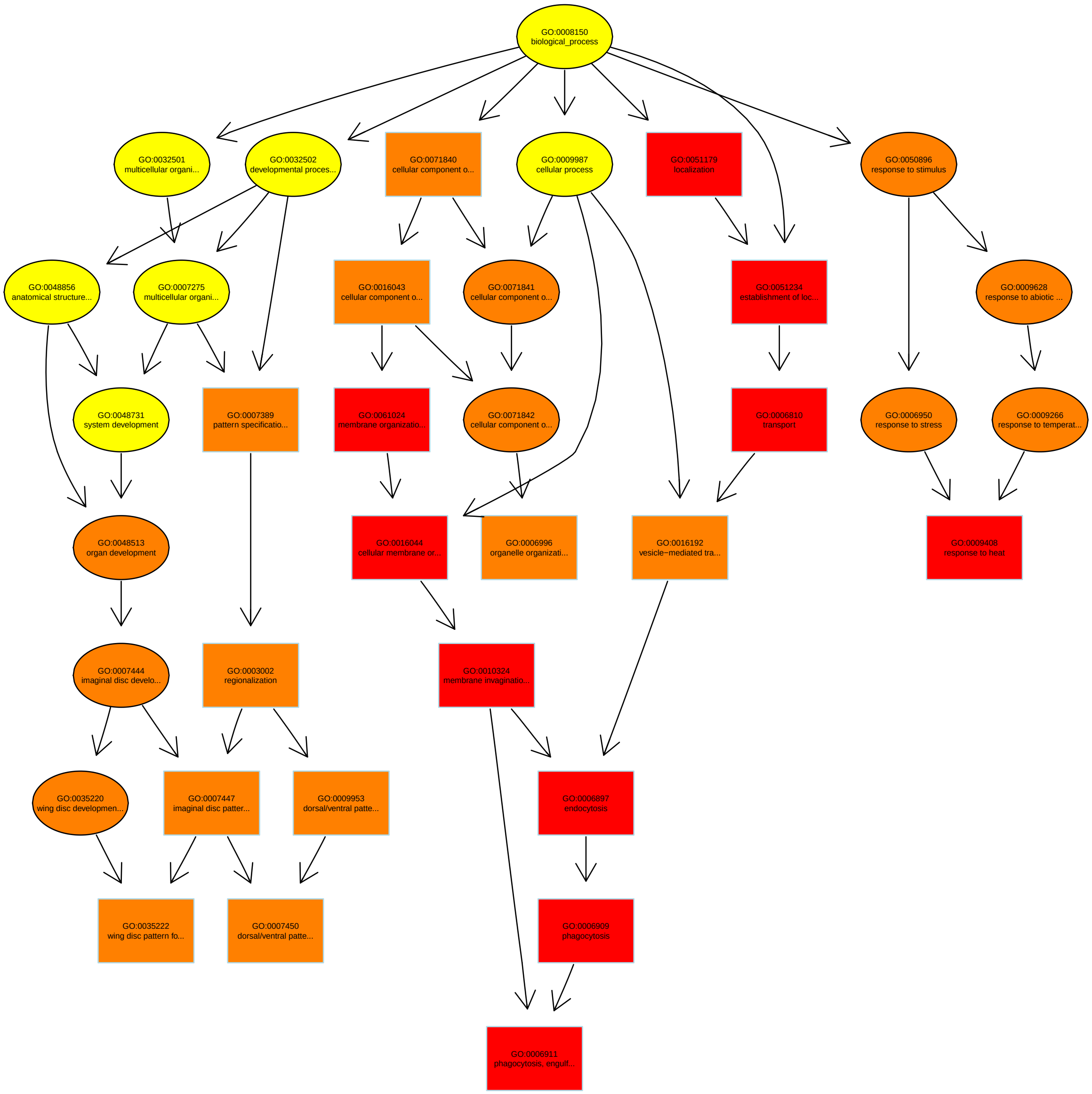

Supplementary Figure S 3: Output of the gene ontology visualizer TopGO for the first use case. Male and female transcriptomes of fruit flies differ (shown in red) mostly in genes related to reproduction and sex determination. These genes are enriched in the ranked list that is the input for this visualizer.

Name and Reference	Input	Output
Read Mapping		
PALMapper†‡ Jean *et al.* (2010)	Index, Reference Genome, FASTQ	BAM
Bowtie− Langmead *et al.* (2009)	Index, Reference Genome, FASTQ	SAM
BWA− Li and Durbin (2010)	Index, Reference Genome, FASTQ	SAM
TopHat− Trapnell *et al.* (2009)	FASTA/Q, Index	BAM, WIG, BED
STAR− Dobin *et al.* (2012)	FASTA/Q, Index, Reference Genome	BAM, BED
Gene and Transcript Prediction		
Cufflinks− Roberts *et al.* (2011)	SAM/BAM, (GFF3)	GTF
mTIM†‡* Görnitz *et al.* (2011b)	FASTA, BAM, SPF	GFF3
Scripture†* Guttman *et al.* (2010)	SAM/BAM	GTF
SplAdder†‡* (in preparation)	FASTA, GFF3, BAM	GFF3
Trinity† Grabherr *et al.* (2011)	FASTQ	FASTA
Quantitative Analysis		
rQuant†‡ Bohnert and Rätsch (2010)	GFF/GTF, BAM	GFF3
rDiff†‡ Stegle *et al.* (2010)	GFF/GTF, BAM	TAB (Gene Names)
Cuffdiff− Roberts *et al.* (2011)	SAM/BAM, (GFF)	GTF
DESeq† Anders and Huber (2010)	GFF/GTF, BAM	TAB (Gene Names)
DESeq2† Anders and Huber (2010)	GFF/GTF, BAM	TAB (Gene Names)
DEXSeq† Anders *et al.* (2012)	GFF/GTF, BAM	TAB (Gene Names)
edgeR† MD *et al.* (2010)	GFF/GTF, BAM	TAB (Gene Names)
GeneSetter†‡	TAB (Gene Names)	PNG, TAB (Percentages)
TopGO† Alexa *et al.* (2006)	TAB (Gene Names)	PDF
Machine Learning-based Sequence Analysis		
KIRMES†‡ Schultheiss *et al.* (2009)	FASTA	PNG, PWM, TAB, HTML
ASP†‡ Sonnenburg *et al.* (2007)	FASTA	GTF
ARTS†‡* Sonnenburg *et al.* (2006)	FASTA	GTF
EasySVM†‡* Ben-Hur *et al.* (2008)	FASTA, ARFF, TAB	TAB (Classifications), PNG
Shogun†‡ Sonnenburg *et al.* (2010)	TAB, Labels	TAB (Classifications)
Pre- and Postprocessing, File Format Utilities		
GFF toolkit† Sreedharan *et al.* (2011)	GFF, GFF3, GTF	GFF3
SAMtools− Li *et al.* (2009)	SAM, BAM	SAM, BAM
RNA-geeq†‡* (in preparation)	GFF3, BAM	BAM, TAB (Score Matrix)
WebLogo†* Crooks *et al.* (2004)	PWM	PNG

Supplementary Table S 1: The software packages integrated into *Oqtans*, together with their respective input and output file formats. For file format abbreviations, see Supplementary Table S2. Packages with an * are currently being updated. Tools for which one of the authors developed a wrapper for *Galaxy* integration are indicated with a †, while tool wrappers developed by others are marked with −. Methods that are developed by one of the authors indicated with a ‡. We intend to include the mGene genome annotation toolbox Schweikert *et al.* (2009) as well, but this is beyond the scope of this work. An up-to-date list of tools including version information is available here: `http://oqtans.org/tools`.

**Extension**	**Stands for**	**Format**	**Used for**
ARFF	Attribute-Relation File Format	Tabular	Databases
BAM	Binary SAM	Binary	Sequence alignment
BED	Browser Extensible Data	Tabular	Sequence annotation
FASTA	FAST-All	Text	Biological sequences
FASTQ	FASTA Quality	Text	Sequence reads with a quality score per base
GFF(3)	Generic Feature Format (version 3)	Tabular	Sequence annotation
GTF	Gene Transfer Format	Tabular	Sequence annotation
HTML	Hypertext Markup Language	Text	Documents with text and graphics
PDF	Portable Document Format	Binary	Layouted text and image data
PNG	Portable Network Graphics	Binary	Image data
PWM	Position Weight Matrix	Tabular	Sequence motifs, e.g. binding sites
SAM	Sequence Alignment/Map	Tabular	Sequence alignment
SPF	Signal Predictor Format	Binary	Trained signal predictors from machine learning methods
TAB	Tabular Values	Tabular	Tabular data, columns separated by a character
WIG	Wiggle	Tabular	Dense continuous data

Supplementary Table S 2: File formats used by the tools described in Supplementary Table S1.

References

Alexa, A., Rahnenführer, J., and Lengauer, T. (2006). Improved scoring of functional groups from gene expression data by decorrelating go graph structure. *Bioinformatics*, **22**(13), 1600–1607.

Anders, S. and Huber, W. (2010). Differential expression analysis for sequence count data. *Genome Biology*, **11**(10), R106.

Anders, S., Reyes, A., and Huber, W. (2012). Detecting differential usage of exons from rna-seq data. *Genome Research*.

Au, K. F., Jiang, H., Lin, L., Xing, Y., and Wong, W. H. (2010). Detection of splice junctions from paired-end rna-seq data by splicemap. *Nucleic Acids Res*, **38**(14), 4570–4578. reads should be >50nt.

Ben-Hur, A., Ong, C. S., Sonnenburg, S., Schölkopf, B., and Rätsch, G. (2008). Support vector machines and kernels for computational biology. *PLoS Comput Biol*, **4**(10), e1000173.

Bohnert, R. and Rätsch, G. (2010). rQuant.web: a tool for RNA-seq-based transcript quantitation. *Nucleic Acids Res*, **38**(Web Server issue), 348–351.

Crooks, G. E., Hon, G., Chandonia, J. M., and Brenner, S. E. (2004). Weblogo: a sequence logo generator. *Genome Res*, **14**(6), 1188–1190.

De Bona, F., Ossowski, S., Schneeberger, K., and Rätsch, G. (2008). Optimal spliced alignments of short sequence reads. *Bioinformatics*, **24**(16), i174–80.

Dobin, A., Davis, C. A., Schlesinger, F., Drenkow, J., Zaleski, C., Jha, S., Batut, P., Chaisson, M., and Gingeras, T. (2012). Star: ultrafast universal rna-seq aligner. *Bioinformatics*, **29**(1), 15–21.

Gan, X., Stegle, O., Behr, J., Steffen, J. G., Drewe, P., Hildebrand, K. L., Lyngsoe, R., Schultheiss, S. J., Osborne, E. J., Sreedharan, V. T., Kahles, A., Bohnert, R., Jean, G., Derwent, P., Kersey, P., Belfield, E. J., Harberd, N. P., Kemen, E., Toomajian, C., Kover, P. X., Clark, R. M., Rätsch, G., and Mott, R. (2011). Multiple reference genomes and transcriptomes for *Arabidopsis thaliana*. *Nature*, **477**(7365), 419–423.

Görnitz, N., Widmer, C. K., Zeller, G., Kahles, A., Sonnenburg, S., and Rätsch, G. (2011a). Hierarchical multitask structured output learning for large-scale sequence segmentation. In J. Shawe-Taylor, R. Zemel, P. Bartlett, F. Pereira, and K. Weinberger, editors, *Advances in Neural Information Processing Systems 24*, pages 2690–2698. Neural Information Processing Systems Foundation, La Jolla, CA, USA.

Görnitz, N., Zeller, G., Behr, J., Kahles, A., Mudrakarta, P., Sonnenburg, S., and Rätsch, G. (2011b). mTiM: margin-based transcript mapping from RNA-seq. In C. Alkan, editor, *RECOMB Sattelite Workshop on Massively Parallel Sequencing*, volume 12, London, UK. BMC Bioinformatics.

Grabherr, M. G., Haas, B. J., Yassour, M., Levin, J. Z., Thompson, D. A., Amit, I., Adiconis, X., Fan, L., Raychowdhury, R., Zeng, Q., Chen, Z., Mauceli, E., Hacohen, N., Gnirke, A., Rhind, N., di Palma, F., Birren, B. W., Nusbaum, C., Lindblad-Toh, K., Friedman, N., and Regev, A. (2011). Full-length transcriptome assembly from rna-seq data without a reference genome. *Nat Biotechnol*, **29**(7), 644–52.

Guttman, M., Garber, M., Levin, J. Z., Donaghey, J., Robinson, J., Adiconis, X., Fan, L., Koziol, M. J., Gnirke, A., Nusbaum, C., Rinn, J. L., Lander, E. S., and Regev, A. (2010). Ab initio reconstruction of cell type-specific transcriptomes in mouse reveals the conserved multi-exonic structure of lincrnas. *Nat Biotechnol*, **28**(5), 503–10.

Jean, G., Kahles, A., Sreedharan, V. T., De Bona, F., and Rätsch, G. (2010). RNA-seq read alignments with PALMapper. *Curr Prot Bioin*, **32**(11).

Lamesch, P., Berardini, T. Z., Li, D., Swarbreck, D., Wilks, C., Sasidharan, R., Muller, R., Dreher, K., Alexander, D. L., Garcia-Hernandez, M., Karthikeyan, A. S., Lee, C. H., Nelson, W. D., Ploetz, L., Singh, S., Wensel, A., and Huala, E. (2012). The arabidopsis information resource (tair): improved gene annotation and new tools. *Nucleic Acids Res*, **40**(Database issue), 1202–1210.

Langmead, B., Trapnell, C., Pop, M., and Salzberg, S. L. (2009). Ultrafast and memory-efficient alignment of short DNA sequences to the human genome. *Genome Biol*, **10**(3).

Li, H. and Durbin, R. (2010). Fast and accurate long-read alignment with Burrows-Wheeler transform. *Bioinformatics*, **26**(5), 589–595.

Li, H., Handsaker, B., Wysoker, A., Fennell, T., Ruan, J., Homer, N., Marth, G., Abecasis, G., and Durbin, R. (2009). The sequence alignment/map format and samtools. *Bioinformatics*, **25**(16), 2078.

MD, R., DJ, M., and GK, S. (2010). edger: a bioconductor package for differential expression analysis of digital gene expression data. *Bioinformatics*, **26**, 139–140.

Roberts, A., Pimentel, H., Trapnell, C., and Pachter, L. (2011). Identification of novel transcripts in annotated genomes using RNA-seq. *Bioinformatics*, **27**, btr355.

Schultheiss, S. J., Busch, W., Lohmann, J. U., Kohlbacher, O., and Rätsch, G. (2009). KIRMES: kernel-based identification of regulatory modules in euchromatic sequences. *Bioinformatics*, **25**(16), 2126–2133.

Schweikert, G., Behr, J., Zien, A., Zeller, G., Ong, C. S., Sonnenburg, S., and Rätsch, G. (2009). mgene.web: a web service for accurate computational gene finding. *Nucleic Acids Res*, **37**(Web Server issue), 312–316.

Sonnenburg, S., Zien, A., and Rätsch, G. (2006). Arts: accurate recognition of transcription starts in human. *Bioinformatics*, **22**(14), e472–80.

Sonnenburg, S., Schweikert, G., Philips, P., Behr, J., and Rätsch, G. (2007). Accurate splice site prediction using support vector machines. *BMC Bioinformatics*, **8**(Suppl. 10), S7.

Sonnenburg, S., Rätsch, G., Henschel, S., Widmer, C., Behr, J., Zien, A., de Bona, F., Binder, A., Gehl, C., and Franc, V. (2010). The shogun machine learning toolbox. *J Mach Learn Res*, **99**, 1799–1802.

Sreedharan, V. T., Behr, J., Bohnert, R., Schultheiss, S. J., and Rätsch, G. (2011). A toolkit for pre-processing genome annotations in generic feature format. In N. Harris and P. Rice, editors, *Bioinformatics Open Source Conference*, volume 12, page 47, Vienna, Austria. Open Bioinformatics Foundation.

Stegle, O., Drewe, P., Bohnert, R., Borgwardt, K., and Rätsch, G. (2010). Statistical tests for detecting differential rna-transcript expression from read counts. *Nature Precedings*, **4437**, 1.

Trapnell, C., Pachter, L., and Salzberg, S. L. (2009). TopHat: discovering splice junctions with RNA-seq. *Bioinformatics*, **25**(9), 1105–1111.

Wang, K., Singh, D., Zeng, Z., Coleman, S. J., Huang, Y., Savich, G. L., He, X., Mieczkowski, P., Grimm, S. A., Perou, C. M., MacLeod, J. N., Chiang, D. Y., Prins, J. F., and Liu, J. (2010). MapSplice: Accurate mapping of RNA-seq reads for splice junction discovery. *Nucleic Acids Research*, **38**(18), e178.
